# Medico-Chirurgical Transactions of Edinburgh, Vol II

**Published:** 1826-10-01

**Authors:** 


*Transactions of the Medico-Chirurgical Society of Edinburgh, instituted,* 1821. Vol. ii. pp. 411, with plates. May, 1826.

WE were quite delighted to see a second volume so soon launched forth by the Northern Medico-Chirurgical Society—a circumstance that evinces great intellectual fertility in the neighbourhood of Salisbury Crags, as compared with Lincoln's-Inn-Fields—Bolt-court—or even the courtly Temple of Apollo, in Pall Mall East. As Helvetia's rugged Alps have been celebrated by the poet for producing abundant crops of—

> " Man and steel—the soldier and his sword ;"

so the flinty-hearted " Auld Reekie," is renowned above all other cities, for her fecundity in conceiving and bringing forth such myriads of grave doctors and learned works that irrigate and fertilize the parched and thirsty intellectual soils of half the world. Well might the Edinburgh school of medicine exclaim

> " Quæ regio in terris nostri non plena laboris ?"

The Northern Council appear to know what they are about. They naturally expect that, among the numerous " tentamina medica" of the modern Athens, some straggling " gems of purest ray serene" must occasionally turn up, whose corruscations may illumine the volumes of their transactions. The thought is a lucky one, and may prove fortunate—at all events, the procedure is perfectly legitimate, and we hope it will conduce to the benefit of both the Council and the authors of the theses. These last are often deterred from publishing in a separate form, by the natural timidity which attends authorship, but, like gregarious animals, they acquire courage when in company with a number of their own species. They are, therefore, under considerable obligations to the Medico-Chirurgical Society of Edinburgh for undertaking the charge of their introduction into public life, without any of those troublesome and expensive circumstances which usually attend the operations of the press, when worked for private use.

The Council are well aware that the greater number of inaugural dissertations are mere compilations of the most superficial kind—that another portion contains little more than juvenile hypotheses—and that very few indeed will bear to be taken from under the protection of their kind patron, that is, the Latin language, to be addressed to the stern public in the mother tongue. Let the Council bear this in mind, and take care how they fish in troubled waters. We shall now proceed to notice the papers seriatim.

### Art. I.

*Account of the Exanthematous Ophthalmia, with Observations on its Treatment.* By James Wardrop, Esq.

In a former paper, Mr. Wardrop observed that there were several distinct species of ophthalmia not accurately described by nosological writers, but which it was important to distinguish. Besides rheumatic inflammation of the eye, there is another, requiring a peculiar treatment, and possessing a distinct character, which has been usually confounded, Mr. W. thinks, with scrofulous inflammation, but which is different therefrom. Mr. W. admits, however, that the exanthematous ophthalmia very often appears in the scrofulous constitution, but only in consequence of such constitutions being more liable to many other diseases. We shall introduce the following graphic delineation of the disease in the words of the ingenious author.

"The species of ophthalmia, to which the term Exanthematous is here meant strictly to apply, is so denominated, from being always either accompanied or preceded by some eruptive disease. Eruptions of the scalp, and discharges behind the ears, so frequent in children, are the affections with which this ophthalmia is most commonly connected. These diseases alternate with the disease of the eyes, the latter becoming affected, when the eruption or discharge disappears; whilst, when either of *these* returns, the eyes recover. This ophthalmia also sometimes succeeds measles, scarlet fever, and other exanthematous diseases, but usually appears a considerable time after these affections have subsided, and when some eruption about the head seems more immediately connected with its appearance.

"The symptoms of the exanthematous ophthalmia are very characteristic; for, besides being connected with eruptions, and confined to young people, the excessive intolerance of light, the enormous secretion of tears, and the relief from forcibly squeezing the eyes, are symptoms quite peculiar. The patient afflicted with this disease, can scarcely hold up his head, and if he is desired to open his eyes, he is affected exactly as if he were looking on a mirror reflecting a bright sunshine, every attempt causing a profuse gush of tears, and being instantly succeeded by a violent and involuntary squeezing of the eye-lids, and knitting of the eye-brows. He excludes all light, not only by holding down his head and squeezing the eye-lids together, but by pressing a handkerchief firmly on them, or by resting his face against a chair in some dark corner of the room. When in bed, he lies with his face buried in the pillow, a circumstance, which of itself points out the peculiarity of this inflammation and distinguishes it from all others.

"The intolerance of light is always most severe in the morning; but in the afternoon it sometimes remits so much, as to allow the patient to open his eyes, and see to a very considerable degree, for some hours. The tears, besides being of an extraordinary quantity, are of an acrid, irritating quality; producing violent paroxysms of sneezing, scalding the cheeks, the alæ of the nose, and the lips; so that these become inflamed and swelled, and sometimes covered with pustules and cutaneous ulcerations. The eye-lids are also swelled, and have turgid veins on their surface. On trying to force them open, a torrent of tears gushes out, and it is not without occasioning great pain, that a small portion of the globe can be exposed. An attempt to get a view of the cornea gives great pain, and it is almost impossible to succeed. The palpebræ, as well as the sclerotic conjunctiva, are but slightly reddened; the vessels appearing as a few distinct trunks, instead of the diffused redness observed in many other inflammations. In general, both eyes are attacked with this disease, though one more violently than the other." 4.

With these local symptoms there is more or less constitutional excitement. The pulse is frequent, the tongue white, the primæ viæ disordered, the abdomen tumid, skin sallow, emaciation. The disease sometimes remains in its aggravated form for many days or weeks—at other times it remits, and the eyes can be opened for a few days, after which it recurs as violent as ever. The constitutional symptoms vary in like proportion; and in this way Mr. Wardrop has known the complaint continue for many months, and even years. When the eruption on the scalp recurs, or the discharge behind the ears increases, the ocular inflammation generally subsides, and the cornea can be examined. Then will be seen a greater or less degree of vascular turgescence in the sclerotic coat, and palpebral conjunctiva, together with one or more specks on the cornea, and even sometimes a distinct ulcer. These, however, are trifling, and indeed there is nothing more remarkable in the history of this disease than the slight injury which the organ sustains, after being long and severely affected. There is no puriform or morbid secretion from the meibomian glands in this inflammation; nor has Mr. W. ever known the structure of any part, except the cornea, permanently changed—a fact which renders it probable, he thinks, that the true seat of the inflammation is in the conjunctiva.

*Treatment.* The general or local depletion, the ointments, opiates, collyria, &c. which relieve some of the other inflammations of the eye, have little or no influence on this species of ophthalmia. And yet it is a disease as much under the control of treatment as most others. Local means, especially in the commencement of the disease, should be confined to cleansing the eyes with warm water, as it will be invariably observed that, as the constitutional ailments abate, the local symptoms will yield, and, in many instances, will be completely removed by the general treatment alone.

"The general treatment which is commonly necessary for the cure of the exanthematous ophthalmia, consists of first completely evacuating the bowels, and afterwards regulating them; of giving alterative and tonic remedies; and of producing an artificial discharge. Even when this ophthalmia appears in a feeble and emaciated child, it will usually be found, that, by the exhibition of purgatives, feculent matter, both unnatural in quantity, and of a bad quality, will be evacuated; and, until its evacuation has been effected, other remedies avail little. One grain of calomel with three of rhubarb, given at bed-time, and repeated every other night, four or five times, whilst jalap or senna is taken on the alternate mornings, will generally answer the purpose of bringing away the feculent contents of the primæ viæ. But whenever the quality of the evacuations improves, these medicines must be given with caution; and one dose of the rhubarb with calomel, given only once in six or eight days, and the senna or jalap occasionally, will be sufficient. For though the greatest benefit will be obtained by evacuating the bowels, violent purging will be found equally prejudicial. When the treatment has been so far advanced, that only one dose of calomel appears necessary in six or eight days, then at this time tonic and stomachic medicines may be advantageously administered. Of these I have found none so generally useful as the carbonates of soda or potass, either given singly, or combined with rhubarb and the bitter infusions. In some instances the mineral acids have been very useful, and also the preparations of iron. Whilst using either of these remedies, much attention is also due to food and habits of life. All wines and malt liquors are particularly hurtful, and the patient should live chiefly on farinaceous vegetables, with but a very small proportion of animal food. The body should not be loaded with clothes, and the head particularly should be slightly covered; protecting the eyes with only a single and narrow fold of black silk, hanging loosely over them, and not wearing a large bonnet. The hair ought to be cut very short, and the greatest advantage will be found from sponging the head and neck with water every morning; using it at first of an agreeable temperature, and making it colder by degrees, particular care being taken to dry the head well afterwards." 8.

In some cases additional means may become necessary, as a succession of blisters behind the ears, or one occasionally to the nape of the neck. Or the antimonial ointment may be substituted, so as to produce a plentiful crop of pustules on the scalp. Frequently, when the intolerance of light and the excessive lachrymation are subdued, the vessels of the palpebral conjunctiva will be found distended with blood. A scratch with a sharp-pointed instrument will give issue to an astonishing quantity of this fluid, with remarkable relief. It may be usefully repeated a few times in such cases. If these scarifications prove insufficient, a little of the red oxide of mercury ointment, introduced within the eye-lids, will be followed by a decided diminution of the symptoms. When specks or ulcerations are perceived on the cornea, the stimulating ointment ought to be re-applied, at intervals of four or eight days, until the transparency is restored. Great care should be taken to prevent relapses, by attention to the functions of the digestive organs, and by keeping up a drain from the neighbourhood of the head by a seton or issue. Country air, if attainable, will be of great advantage.

We need hardly say that this paper of Mr. Wardrop's will prove interesting to the medical practitioner, and is creditable to the acute observation and accurate discrimination of the author.

### Art. II.

*Observations on the Nature, Causes, and Treatment of Beriberi.* By William Hamilton, Esq. Surgeon, H.E.I.C.S.

The singular disease which forms the subject of Mr. Hamilton's communication, is principally, if not entirely, confined to the Island of Ceylon, the Malabar coast, and that tract of country stretching from Madras to Ganjam in length, and about forty miles in breadth. It is most prevalent during the decline of one monsoon and setting in of another, "when the atmosphere is completely loaded with cold, raw, damp vapours, and the vicissitudes of temperature are greater than at any other period of the year." It seldom occurs at a distance exceeding sixty miles from the sea. A residence of some months in the locality where the disease prevails, seems necessary to its production. There is no constitution exempted from an attack of Beriberi; but those who are sedentary, debauched, and much exposed to vicissitudes of weather are most subject to the disease. One attack also leaves a predisposition to another. The old and infirm are more liable than the young and active.

Mr. Hamilton had an opportunity of seeing the complaint under two forms only, viz. that in which the symptoms were at first mild, and gradually increased in severity—and that in which the symptoms were, even from the first, rather urgent, increased rapidly, and, unless speedily relieved, proved fatal.

The two first cases which fell under Mr. Hamilton's care were treated on the plan recommended by Dr. Christie, (calomel, squills, and other diuretics) but both terminated unfortunately. Mr. H. now examined the body of the second patient, and found an ounce of serum effused between the pia mater and tunica arachnoidea, with two or three dark red patches, exceedingly vascular, extending into the substance of the brain. There was also effusion into the ventricles, and at the base of the brain. The lungs were loaded with dark-coloured blood, and water was effused into both cavities of the chest. The heart was healthy, no effusion into the pericardium, but this bag exhibited marks of inflammation both internally and externally. The liver was larger than natural, and appeared still more loaded than the lungs. On examining the spinal marrow, evident marks of congestion were found, particularly in the dorsal region. Three or four pints of effusion had taken place in the abdomen.

These post mortem appearances coincided a good deal with those published by Mr. Ridley, in the Dublin Hospital Reports, and determined Mr. Hamilton as to the nature of the disease. He became convinced that it arose, in a great measure, from obstructed circulation, in consequence of congestion in the internal parts, more especially the liver and lungs, and that beriberi consequently could not be a mere disease of debility, as supposed by Colquhoun, Hunter, Christie, and others—but, on the other hand, that it was a disease, in the treatment of which, blood-letting might be used with the greatest prospect of advantage.

"The prevalence of the disease," (says Mr. H.) "during the change of the monsoons, may be accounted for, by the damp, loaded state of atmosphere, and the extreme vicissitudes of temperature, which, by suddenly checking perspiration and producing (to use the words of Dr. Johnson in his very excellent Essay on Cholera) 'unparalleled atony of the extreme vessels, debilitated by previous excess of action, break at once, and with violence, the balance of the circulation. The extreme vessels of the hepatic system sympathising with those on the surface, completely arrest the reflux of blood from the portal, cœliac, and mesenteric circles;' hence that gorged state of the internal parts, which appeared in the case I have related, and which, in a still more marked degree, is found to exist in the cholera of India." 21.

From these considerations, Mr. Hamilton resolved to employ venæsection in the subsequent cases that fell under his care. He, therefore, abstracted 30 ounces of blood from the next patient, which gave immediate though temporary relief, and encouraged him to proceed to the abstraction of 35 ounces more, in the course of the next twelve hours, the dyspnœa and other symptoms having returned. Immediate recourse was now had to mercury, on which Mr. H. confidently relied, if he could bring the system under its influence. He accordingly directed 20 grains of calomel with 30 drops of laudanum, to be given, and a vapour bath to be applied. In an hour and ten minutes the calomel was repeated, with the laudanum, soon after which the patient fell into a sound sleep, and awoke in a copious perspiration. His pulse had now increased in strength, and the dyspnœa was not near so distressing. The calomel was repeated, with gamboge, and afterwards the calomel alone, in scruple doses, every three or four hours, until ptyalism was established, which required more than forty hours. Every unfavourable symptom now disappeared, and the patient complained only of soreness of mouth.

In another case, there was, from the first, violent and continued vomiting, which yielded, however, to large and repeated doses of calomel and laudanum, with a strong and heated sinapism to the region of the stomach—a remedy which Mr. H. found singularly successful in speedily allaying the violent gastric irritability of bilious remittent fever and cholera, where calomel and laudanum had failed.

In one case, where the individual was of rather a full habit, bleeding was thrice employed, the patient losing, in all, more than 65 ounces of blood, within thirty hours—"and that with the happiest effects." This practice was adopted too, although the dropsical and other symptoms had existed for nearly two days before Mr. H. saw the patient. The quantity of blood to be drawn must, of course, be regulated by the symptoms in every particular case, as well as by the age and constitution of the individual.

Dr. C. Rogers, who practised for some time in Ceylon, has communicated to Mr. Hamilton the particulars of two cases treated by blood-letting, in which there was a striking alleviation of all the symptoms, as soon as blood was abstracted. With the following extract from Mr. Hamilton's paper, we may conclude this short notice.

"In cases where there exists irritability of the stomach, or bowels, or of both conjoined, as is very frequently the case in some of the diseases of India, or where my object is the speedy production of ptyalism, I never think of exhibiting calomel in smaller doses than from 15 to 20 grains—and in the propriety of this I am confirmed by the experience of others. In such doses it seems to act as a sedative, in so far as it allays vomiting, removes griping and spasm, and frequently procures for the patient sound sleep; while in smaller doses, as from four to seven and even ten grains, it operates as a purgative, often producing considerable griping, with sickness and a general sense of lassitude. Experience has likewise established the importance of this medicine being given in the form of powder instead of pills, particularly in cases where the bowels are much affected, as in dysentery or cholera morbus; for in such cases pills have been frequently known to pass off nearly as they had been given." 28.

One case is given in detail, but it is unnecessary to state any of the particulars in this place.

### Art. III.

*Description of two Children united together, and* now living *in the Village of Arasoor*. By Andrew Berry, M.D. With a Plate.

According to the title of this paper, and the date of Dr. Berry's letter, (Jan. 1824,) these children should now be upwards of twenty years of age, and still living; but, in the body of the communication, we find they died nearly fifteen years ago. We are not very partial to the recital or record of monstrous deviations from the general form or structure of the human body, as they seldom lead to any useful conclusion, physiological or otherwise;—but as human curiosity must be gratified occasionally by some marvellous tale, we beg to inform all our curious readers, that these twin sisters were brought forth without any difficulty—that they were connected by their sternums—that they had only one umbilicus—that one of them was entirely nourished, for some months after birth, "by what it received from the stomach or other part of the alimentary canal of its sister"—and that, finally, one of them died when nearly seven years of age, which necessarily proved fatal to the other. No dissection took place, and consequently we are left in ignorance of the precise nature of the malformation which united these children.

Immediately in succession, is a short account, by Dr. Hastings, of another monster, in whom the upper and lower extremities were entirely wanting. The cupidity of the parents proved destructive to the offspring, and subversive of the object they had in view. The child was seized with pneumonia, while hawked about for exhibition at fairs and markets, and died. We see nothing worthy of notice in the dissection which Dr. Hastings made of this memberless trunk. There are drawings of this and the preceding monster. These drawings have enhanced the expense, without adding to the value of the volume.

### ART. IV.

*Observations on Chronic Inflammation of the Iris.* By ALEX. WATSON, Esq.

In this disease, which is of very slow progress, effusion of lymph on the capsule of the lens and inner surface of the cornea, adhesion of the iris, and contraction of the pupil, frequently take place to such an extent as to cause complete blindness. The following is Mr. Watson's description of the disease, omitting his references to the figures in the plate which accompanies the communication.

"In this disease, the first change observed in the eye, is a partial irregularity of the pupillar margin of the iris, at one or more points. This irregularity of the margin of the iris, alters, of course, the form as well as the size of the pupil. In some cases, it is more dilated, and in others more contracted, than natural. The iris loses its proper colour; and its pupillar margin becomes partially or wholly drawn backwards, in consequence of its partial or complete adhesion to the capsule of the crystalline lens. The motions of the pupil, at the same time, become impeded, in proportion to the number and extent of the adhesions. In some cases, the adhesion of the iris takes place to a considerable extent at one point; in other cases, the adhesion takes place to a smaller extent, at several points, which, in the progress of the affection, by extending, become one continued adhesion, involving the greater part of the whole of the pupil. That part of the iris between the adhesion and the ciliary margin, assumes a convex form, by projecting to a greater degree, at this part, towards the cornea, than it does in its healthy state. By this projection, the size of the anterior chamber of the aqueous humour is diminished, and that of the posterior chamber is proportionably increased.

"Where the iris adheres to the capsule of the lens, an effusion of lymph may, in general, be observed, forming the connecting medium. The capsule of the lens generally becomes opaque; and frequently small portions of lymph, and sometimes of pigment, from the posterior surface of the iris, can be seen upon this capsule. In some cases a deposition of lymph takes place upon the inner surface of the cornea, occasioning a dimness and opacity of this part. Vision gradually becomes impaired, as the disease advances, till it is quite destroyed. And this last symptom (impaired vision) is commonly the only one by which the patient is conscious that mischief is going on in the eye." 44.

It is to be regretted that nothing satisfactory can be said of the treatment. Mercury failed to cure the disease—and so, we suppose, did every other medicine, since Mr. Watson does not mention any.

ART. V.

*Account of the Yellow Fever, as it appeared in the Queen's Regiment in Barbadoes, in* 1816 *and* 1817. By ALEXANDER RALPH, M.D. Assistant-Surgeon to the Regiment.

This is one of the three inaugural dissertations introduced into this volume of Transactions. However faithful may be the narrative, however accurate the descriptions, which Dr. Ralph has laid before us of this fearful scourge, still it must be acknowledged that the subject of yellow fever is now more than a thrice told tale, and that Dr. Ralph's paper presents nothing which has not been reiterated repeatedly by former writers. Still it contains many judicious observations and acute remarks on the causes, course, and treatment of yellow fever, which must prove interesting to those whose fortunes or misfortunes lead them to the scene of its devastations. We were much pleased to find Dr. Ralph doing justice to the venerable and truly philanthropic Jackson, whose writings have not been duly appreciated by the profession in this and other countries. They contain, along with some peculiar, perhaps erroneous notions, the best description of West Indian fevers that has ever been published—and the truest pathology of those merciless endemics.

ART. VI.

*Observations on the prevailing Opinions respecting Respiration and Animal Heat; with Experiments.* By C. S. B. WILLIAMS, M.D.

This is the second "tentamen" which the Council has selected for publication; but, being a purely physiological discussion, and consisting chiefly of comments on the opinions and doctrines of others, we are unable to afford space for any analysis of it here. Dr. Williams has fairly stated the merits and defects of the two most prevailing theories respecting respiration in this country, and made some judicious remarks of his own. This paper will be read with interest by most of the junior members of medical society, to whom physiology is always more alluring than therapeutics, their minds being less distracted by the multifarious concerns of active practice than those of their elder brethren. Dr. Williams appears to consider animal heat as the result of neither purely chemical changes nor vital actions—but as the consequence of both combined.

"From a consideration of all the facts which I have now stated, I am led to the belief that animal heat is the result of chemical changes proceeding in the blood, and which I have endeavoured to particularise as those resulting from the functions of *respiration and secretion*, and that a due performance of these functions is requisite for the healthy and uniform preservation of animal temperature." 116.

### Art. VII.

*Case of Extraction of Calculus from the female Bladder, by Dilatation of the Urethra.* By Robert Hamilton, M.D. Secretary.

The very few trials which have yet been made to dilate the female urethra, for the extraction of calculi, have not sufficiently decided the superiority of the sudden or the slow mode of procedure. The following case is brought forward by Dr. Hamilton with the view of helping to decide this question.

*Case.* A hale, stout woman presented herself at the New Town Infirmary, labouring under symptoms of stone, of six or eight years' duration. She was examined, ordered some opening medicine, and next day Dr. H. introduced Weiss's dilator turning up the screw two teeth. In an hour, much pain had been produced, and some drops of blood oozed from the urethra. The screw was turned another point, with great increase of suffering. An opiate given. In an hour and a half, these sufferings had increased much, and more blood oozed from the meatus. The screw was turned to the 4th division on the scale, and afterwards, at short intervals, two points more were turned, when the diameter of the passage was dilated to the extent of three quarters of an inch. The whole had occupied six hours. The instrument was withdrawn, and the forceps easily introduced. The stone was grasped, but could not be brought away. Dilatation was again effected, and the screw raised to the 7th point on the scale. An opiate was administered, and the dilator left in for the night, which was passed in great torture, and without any sleep. Next morning the screw was raised to the 9th point, making the bore of the meatus $1\frac{1}{8}$. The patience of the woman was now exhausted, and extraction was again attempted, but without success; —the stone being too large to come through the dilated passage. The bistoury was therefore introduced, and Dr. H. cut at the angle formed by the junction of the bladder and urethra, in a direction upwards and outwards. The bridle which had obstructed the progress of the stone was thus divided, and the calculus was easily extracted. No inconvenience followed, and the woman was soon able to return to her avocations. The stone weighed an ounce, and measured four inches and a half in its long circumference, by one and a quarter in its short. Eighteen hours had been expended in the dilatation. Dr. H. comes to the conclusion, from this case, "that, when a stone is large, it will not, in all cases, be possible, with the utmost care, to effect the necessary dilatation in a short space of time, and that the more gradual dilatation, carried on for several days, by means of the sponge tent, will prove safer and easier than the more rapid operation of the new invented dilator." As far as one case can go, Dr. H. is borne out in this conclusion. But the stone was very large in this instance—and, at all events, it is but a single case.

### Art. VIII.

*Case in which a Calculus of considerable size was extracted from the Female Bladder, by gradual dilatation of the Urethra.* By Alexander Ramsay, M.D. of Dundee.

This was a lady, 67 years of age, who had been afflicted with symptoms of calculus for 20 or 30 years. These symptoms had disappeared for a time, and afterwards returned; and at the period when Dr. Ramsay was consulted (1822) the constitution had sympathised with the local irritation to an alarming extent. On sounding, Dr. R. discovered a stone of some magnitude. The vagina was contracted, with some thickening and induration around the urethra, neck of the bladder, and rectum. The cutting operation was, under all circumstances, deemed unadvisable, and dilatation was readily submitted to by the patient.

Two weeks were spent, with very partial success, in dilating the urethra by sponge tents, which were generally expelled in a few hours after their introduction, but sometimes remained a whole night, though not without distress. Weiss's dilator was found much more effectual than the tents, and Dr. R. succeeded in expanding the urethra more and more every day, from 20 to 30 minutes at a time, until, in the course of ten days, he could easily introduce the fore-finger into the urethra, and examine the calculus. After a considerable dilatation, the forceps was introduced, and fortunately the stone was seized in a good position, and extracted, with some difficulty, and a good deal of pain to the patient. The chief resistance was experienced at the orifice of the urethra. The long circumference of the stone was 5½ inches, by 3½ in the short. It weighed 7½ drachms. Dr. R. thinks it would have been impossible to have extracted the stone in this way through the urethra, had not the dilatation been effected in a very gradual but decided manner for some weeks previous to the operation. A good deal of constitutional irritation and local inflammation followed the extraction, but these subsided, and the functions of the bladder and urethra were soon restored. The old lady now enjoys good health.

### Art. IX.

*Cases illustrating the Contagious Nature of Erysipelas, and its Connexion with a Severe Affection of the Throat.* By John Stevenson, M.D. of Arbroath.

The occasionally contagious character which erysipelas assumes, is well known to all who have paid any attention to the complaint in the wards of hospitals; but its connexion with an affection of the throat has not, as far as we remember, been particularly noticed by systematic writers, though many instances are related by individuals, where the inflammation appeared to spread from the fauces to the external integuments. This affection of the throat occurred so frequently in persons who had been much with erysipelatous patients, that Dr. S. could not doubt their identity; and he came to the conclusion that it was, in reality, erysipelas of the fauces, spreading occasionally to the adjacent parts in different directions. The febrile symptoms by which the complaint was ushered in were generally severe, even in the milder cases—pulse full and frequent—severe pain of head and back—restlessness and great heat of surface. The affection of the throat came on at periods varying from two to six days after the accession of the fever.

"It commonly began with a red or purplish blush, more or less extensive, over the velum pendulum and uvula, accompanied with very little tumefaction, but with considerable pain in swallowing; often, after a few days, excoriation of the inflamed surface followed, with superficial ulceration, which at times soon healed, but at other times spread and discharged a good deal of purulent matter. In many cases the disease terminated without extending farther than the parts mentioned, but in a few it spread to the larynx, producing a state of respiration very like that of idiopathic croup; in others it extended to the pharynx and œsophagus. When the last became affected, fluids and even solids could be partially swallowed without much apparent difficulty; but after a few seconds, pain was felt in the course of the gullet, an inverted action began, and they were wholly or partially returned to the mouth. In some protracted cases glandular swellings appeared in the neck, which suppurated externally." 129.

The disease was readily distinguished from cynanche tonsillaris, by the want of swelling, by the redness being more diffused, and by the pyrexia being generally greater than could have been expected from the degree of local affection. From croup it was distinguishable by the larynx being affected in a small proportion only of the cases, by the inflammation not commencing there, and by the age of the patients. From scarlatina it was distinguishable by the absence of cutaneous eruption, and by its attacking persons who already had had that disease.

"Copious and repeated bleeding, with brisk purgatives, and, in every case where the throat was severely affected, the application of a large number of leeches to the neck, appeared to me to be the mode of treatment which was most successful. All the cases that came under my management, both of the common erysipelas, and of the affection of the throat, were treated in this manner. I have stated the event of all those detailed, and it affords a fair specimen of the general results of the cases which came under my care," 130.

A considerable number of cases, in a very brief form, are appended to this account, all of which recovered. These we need not particularise in this place. But we may remark that, although the treatment detailed above was very proper and successful in Arbroath, it is by no means to be taken as a model for our imitation. Even in the same locality, we much doubt whether this mode of treatment would always succeed; but in London, for instance, and in large manufacturing towns, depletion, in every form of erysipelas, must be employed with caution, especially among those whose constitutions are deteriorated.

### Art. X.

### *A Case of Mollities Ossium, with the Appearances on Dissection.* By John Howship, Esq.

This case, whose "lengthened course extended almost to the space of six years," was attended with an infinite number of curious and anomalous symptoms, the like of which we shall seldom meet again. We wish we could believe with the ingenious narrator, that it developed "certain principles by which the treatment of the disease should be regulated, and by which the previous disposition may, on future occasions, be treated with advantage and success." We shall find it rather a difficult matter to compress within our usual limits the history of this lady's afflicting and fatal malady, but we shall endeavour to do so, if possible.

*Case.* Miss H. aged 35 years, while visiting Paris in 1816, had got cold, but returned to England in tolerable health. Soon after this she was observed to stoop in her gait, and walk in a rolling manner. An eruption broke out on her head, and Mr. Thomas ordered her decoction of oak bark, by a perseverance in which "she seemed to come round," but "still, however, she declined." Her appetite was bad. In 1818, being much worse, Dr. L. was consulted—considered the complaint as one of disordered liver, and ordered mercury, under the influence of which the patient got worse. A temporary residence in Staffordshire, without any medecine, improved her health much, but she still walked ill, and was obliged to use a staff. She had pain in her right hip, which was much increased by a cough. In walking, she was afraid of the least pebble in the street touching the right foot. In the autumn of this year she went to Margate quite a cripple—used the warm sea-bath twice a week—and returned surprisingly better. In London, she soon fell off again, and got into a state of bad health. While riding in a carriage one day in 1821, the pain in the hip came on in such a violent manner that she was obliged to be carried to her bed-chamber, where she remained till her death. The late Mr. W. (Wilson we suppose) was now consulted, and thought that a curvature was taking place in the spine, for which a seton was inserted, and kept open five months. The pain in the right hip still continued to be most severe—"as if being torn all to pieces." She got weaker, and the seton was at length removed. In August, 1821, she consulted another surgeon, who recommended the horizontal position without change. One night, while being shifted, she exclaimed that her leg was broken, and remained in great pain all night. Mr. W. was summoned, and, on examination, believed that fracture had actually taken place. "The limb laid in a state of quietude and repose, had, after this accident, remained three weeks at liberty, when she awoke early one morning, crying out in dreadful agony, which, if it continued, she said, must drive her distracted." The middle part of the right thigh was now observed to be raised up "like an elbow," the muscles surrounding the limb being affected with cramps and spasms, and "the whole frightfully painful."—Next day Mr. W. laid the thigh straight and secured it with splints. About a month after this the *left* thigh became very painful; one night the femur gave way, like the other, and Mr. W. found one end of the bone riding over the other, the next day. This limb was now also set in splints. It was in the December of 1821, that Mr. Howship first saw the patient, and finding her both weak and exceedingly irritable, he ordered bark and saline aperients. Her digestion improved a little, but pains came in the left arm, of a very distressing kind. Her nervous excitability was such that the very action of swallowing induced violent pain in the diseased members—and so did the slightest touch of a cambric handkerchief on the face—nay, even the mental emotion incident to speaking of her complaints, would produce paroxysms of pain.

On the 16th December, Mr. H. examined both thighs, when the following extraordinary phenomenon was observed.

"The skin was moist and clammy, but extreme apprehension of suffering on their exposure, induced her to intreat that they might not be touched; so that the degree of firmness the bones might possess could not be ascertained. Upon passing my finger along the surface of the integuments of the thigh, large drops of perspiration were immediately seen to start out along the lines, and there only; an experiment I several times repeated, and always with the same result. Neither was the fluid excreted less singular, possessing, as it did, a strong, very unpleasant, and most unusual odour." 146.

Another curious circumstance was, that touching one limb excited uneasiness in the other, and *vice versâ*, while no distress was felt in the limb that was handled. And now she was annoyed with a sensation in each femur, "as if a string was tied tight round the middle of the bone," a pain which was afterwards accounted for in a very satisfactory manner. Various tonics were prescribed, which sometimes appeared to do good, but were often interrupted by some inflammatory affection of the chest. We cannot follow Mr. Howship very closely in his detail of symptoms in this case. We find, in the month of May 1822, that, on examination, the bone of the left thigh was completely flexible—in short, that there was mollities ossium. On the sixth of the next month death put a period to this lady's sufferings.

*Dissection.* The left thigh was opened first. The muscles were not much wasted, and the fat was abundant.

"Laying aside the rectus femoris, so as to expose the cruræus, the parts immediately beneath that muscle felt like an enlargement of what seemed to lie in the line of the femur. The tough yet thin membrane of the periosteum was apparently filled with some soft or fluid matter. The periosteum being longitudinally divided, the contents proved to be a red pulpy or fleshy matter, in some parts much resembling liver; in one place much softer; in another of a grumous consistence like blood. The whole of the softened femur, removed from its place, admitted of a perfect longitudinal division by the knife through the cylindrical portion, without its meeting with the least trace of ossific matter; but towards each extremity it occasionally encountered a few scattered spiculæ of bone, or a thin external lamina, like a small fragment of paper, or egg-shell." 153.

Although the medullary secretion was every where deranged the matter deposited was by no means uniform in appearance. One mass resembled pure coagulated blood; another, gorged liver, and a third had the appearance of compact fleshy substance. The periosteum was not materially thickened; but

Mr. H. found that precisely in the point to which, during life, the peculiar sense of stricture had been referred, a stricture actually existed. The periosteum at this part had precisely the appearance that would have been produced by a ligature tied tight round it. The right femur presented the same phenomenon, and the other morbid structure described. The bones of the pelvis were so nearly destroyed as to admit of being cut through at pleasure. The viscera presented no morbid appearance.

Such is the history, and such the *post mortem* inspection of this case. Excepting the great and sudden amelioration experienced, in an early period of the complaint, by a temporary residence and some warm-baths on the coast, we cannot see any thing that materially tended to arrest the progress of the malady. True it is that, whether the constitutional predisposition or rather indisposition be likely to lead to morbid softening or morbid hardening of bone or other structure in the body, we shall often find a "trip to Margate," and the salt-water baths there almost realize the fable of Medea's Cauldron; and, therefore, we are strong advocates for this measure whenever hygiene is likely to be more beneficial than therapeutics. Farther than this we do not see in what respect the present case has enlarged our knowledge of either the nature or the treatment of mollities ossium.

In our next, we shall continue the review of several other articles in this volume, some of which are of very considerable interest and importance.

